# Blood-Based Biomarkers of Repetitive, Subconcussive Blast Overpressure Exposure in the Training Environment: A Pilot Study

**DOI:** 10.1089/neur.2022.0029

**Published:** 2022-10-31

**Authors:** Denes V. Agoston, Jesse McCullough, Roxanne Aniceto, I-Hsuan Lin, Alaa Kamnaksh, Michael Eklund, Wallace M. Graves, Cyrus Dunbar, James Engall, Eric B. Schneider, Fabio Leonessa, Josh L. Duckworth

**Affiliations:** ^1^ Department of Anatomy, Physiology, and Genetics, Uniformed Services University, Bethesda, Maryland, USA.; ^2^ NeuroTactical Research Team, Marine Corps Base Camp Pendleton, Camp Pendleton, California, USA.; ^3^ Department of Surgery, Yale School of Medicine, New Haven, Connecticut, USA.; ^4^ Department of Neurology, Uniformed Services University, Bethesda, Maryland, USA.

**Keywords:** biomarker, blood, heavy, protein, training, weapons

## Abstract

Because of their unknown long-term effects, repeated mild traumatic brain injuries (TBIs), including the low, subconcussive ones, represent a specific challenge to healthcare systems. It has been hypothesized that they can have a cumulative effect, and they may cause molecular changes that can lead to chronic degenerative processes. Military personnel are especially vulnerable to consequences of subconcussive TBIs because their training involves repeated exposures to mild explosive blasts. In this pilot study, we collected blood samples at baseline, 6 h, 24 h, 72 h, 2 weeks, and 3 months after heavy weapons training from students and instructors who were exposed to repeated subconcussive blasts. Samples were analyzed using the reverse and forward phase protein microarray platforms. We detected elevated serum levels of glial fibrillary acidic protein, ubiquitin C-terminal hydrolase L1 (UCH-L1), nicotinic alpha 7 subunit (CHRNA7), occludin (OCLN), claudin-5 (CLDN5), matrix metalloprotease 9 (MMP9), and intereukin-6 (IL-6). Importantly, serum levels of most of the tested protein biomarkers were the highest at 3 months after exposures. We also detected elevated autoantibody titers of proteins related to vascular and neuroglia-specific proteins at 3 months after exposures as compared to baseline levels. These findings suggest that repeated exposures to subconcussive blasts can induce molecular changes indicating not only neuron and glia damage, but also vascular changes and inflammation that are detectable for at least 3 months after exposures whereas elevated titers of autoantibodies against vascular and neuroglia-specific proteins can indicate an autoimmune process.

## Introduction

Studies on the effects of repeated, subconcussive traumatic brain injuries (TBIs) in collision sports (e.g., boxing, football, hockey, and soccer) have shown transiently impaired neurocognitive and -motor performance.^1^ However, in a minority of persons, it could initiate degenerative conditions like chronic traumatic encephalopathy (CTE), but the pathobiological process is poorly understood.^2^ Importantly, there is the indication of a dose-response relationship between cumulative TBIs and later-in-life neuropsychiatric abnormalities.^3^ Longitudinal studies with athletes have shown microstructural changes in the brain, including increased white matter diffusivity, altered functional connectivity of the default mode network, reductions in cerebrovascular reactivity, and, on a longer time scale, even reductions in brain volume.^4^

Multiple studies have shown that concussion, especially repeated concussive TBIs including—importantly—repeated exposures to low-level explosive blasts, can cause neuron, astroglia, and vascular injury, as well as neuroinflammation as reflected by elevated serum levels of related protein biomarkers.^5–8^ ^9,10^ Injured astroglia and neurons release cell-type–specific markers, such as glial fibrillary acidic protein (GFAP) and ubiquitin C-terminal hydrolase L1 (UCH-L1)^11^ and neuronal subtype-specific markers, like nicotinic alpha 7 subunit (CHRNA7), that mediate cholinergic transmission.^12^ Elevated serum levels of endothelial tight junction proteins occludin (OCLN) and claudin-5 (CLDN5) indicate vascular injury.^13^ Matrix metalloprotease 9 (MMP9), involved in tissue remodeling after physical insults and the inflammatory response to injury,^14^ has been found to be elevated in serum after TBI.^15^ Intereukin-6 (IL-6), a proinflammatory cytokine,^16^ has also been found to be elevated in serum after experimental blast TBI^17^ and after repeated exposures to low-level of explosive blasts.^5^

In addition to the involvement of the innate immune system in the initial response to TBI, there is also evidence for an adaptive immune response. Studies have demonstrated the presence of autoantibodies against several brain-specific proteins, including GFAP, and neurotransmitter receptors after TBI. Autoantibodies against alpha-synuclein (a-SYN), a neuronal protein, were found in patients with neurodegenerative conditions, primarily Parkinson's disease.^18^ Whereas autoantibodies against CLDN5 may indicate altered blood–brain barrier (BBB) functions,^18^ autoantibodies against intracellular adhesion molecule 1 (ICAM-1) and myelin basic protein (MBP) have been found in vascular and demyelinating diseases, respectively.^19^

Training-associated blast exposure, caused by heavy weapons training (HWT) and breaching exercises, is endemic in the military population. Students (trainees) enrolled in HWT courses are regularly exposed to repetitive subconcussive blast exposure events (RSCBEs) over the course of 2–3 weeks. Moreover, instructors (range safety officers) experience an especially high number of RSCBEs during their 2- to 3-year assignments. Over the past decade, the research, operational, and medical communities have become increasingly aware that RSCBEs cause decreased neurocognitive functioning and subjective symptoms (i.e., headache, memory loss, changes in mood, inability to sleep, and balance problems),^20^ but the long-term effects of RSCBEs are largely unknown. Whereas neuropathological evidence of CTE was infrequently found in brains of military personnel,^21^ a unique neuropathology associated with blast was described.^22^ Experimental studies have shown that exposure to explosive blast, including repeated exposure to low levels of blast, causes neuron, astroglia, and vascular injury, as well as neuroinflammation, as reflected by elevated serum levels of related protein biomarkers.^23,24^

Considering the cumulative nature and long-term physiological and neuropsychiatric consequences of repeated subconcussive blast exposure, it is imperative to identify biomarkers for early detection and diagnosis. Therefore, the objective of this pilot study was to determine the feasibility of using blood-based protein biomarker analysis to assess the short- and long-term effects of RSCBEs.

## Methods

Subjects were service members participating in HWT events in San Diego, California, either as students or instructors. Study inclusion/exclusion criteria: male subjects, ≥18 years, who reported no history of moderate-to-severe TBI^25^ and were scheduled to complete a combat training course that included the firing of heavy weapons, either as students (trainees) or instructors (supervisors). Protocol NEU-92-1913—“Investigating the Neurologic Effects of Training Associated Blast (I-TAB)”—was approved by USUHS IRB on January 6, 2016.

**FIG. 1.**
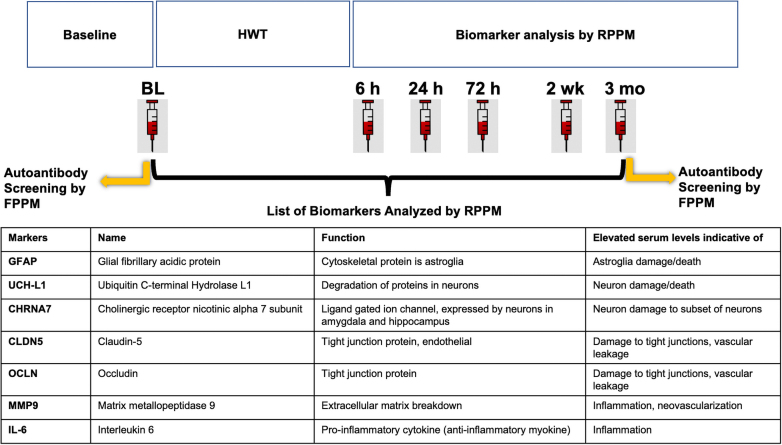
Overall study design of I-TAB, blood sampling time points for biomarker analysis and autoantibody screening, and the list of protein biomarkers analyzed in this study. HWT, heavy weapons training; FPPM, forward phase protein microarray; RPPM, reverse phase protein microarray.

Generally, an HWT session involves six firings of shoulder-mounted weapons for students and three times that amount for instructors. Blast exposure was measured using wearable wireless blast sensors (Black Box Biometrics, Inc., Rochester, NY) and checked for evidence of blast exposure at each assessment time. But overpressure exposure data were not available for this preliminary analysis of biomarker levels. Blood samples were collected before (baseline) and at 6 h (range, ±2), 24 h (±6), 72 h (±12 h), 2 weeks (±3 days), and 3 months (±7 days) after the referenced training event (Fig. 1). For this pilot biomarker study, data from a subset of participants (students = 6; instructors = 10) who were the first to be enrolled in the overall study were assessed. For autoantibody screening, we had access to both baseline and 3-month post-training blood samples from only 10 subjects. Given that this pilot study was conducted to examine the practicality and feasibility of obtaining and assessing repeated measures of select blood-borne biomarkers, in the context of blast overpressure exposure in a real military training environment, no power calculation was performed beforehand.

### Blood collection and preparation of serum samples

Whole blood was collected on-site using standard phlebotomy procedures into BD Vacutainer SST™ II Advance tubes for serum preparation. Tubes were inverted several times and allowed to clot at room temperature for 30 min before centrifugation at 1500*g* for 10 min to separate serum. Sera were then divided into 0.5-mL aliquots, flash-frozen, and stored at −80°C until shipping for subsequent analysis.

### Protein biomarker analysis

Serum samples were analyzed using reverse phase protein microarray (RPPM), a high-sensitivity, high-throughput antibody-based analytical platform.^17,23,24,26-41^ Briefly, samples were denatured and loaded into each well in a 96-well plate and serially diluted in a 1:2 manner (five-step) to yield six total sample concentrations. Plates were then loaded into a PerkinElmer liquid handling robot (Janus 3), which transferred the serially diluted samples to the 384-well source plates in a pre-determined layout. Source plates were moved into an Aushon 2470 Arrayer (Quanterix, Billerica, MA), and samples were printed onto ONCYTE AVID nitrocellulose film slides. Printed slides were air-dried, then washed. Primary antibodies were validated by the conventional western blotting technique for specificity; after quality control^26^ slides were incubated with the primary antibodies (see Supplementary Table S1 for antibody product and dilution details) overnight (8–12 h) at 4°C and then washed. Slides were incubated with their respective secondary antibody solutions, washed and dried, and then loaded into an Innopsys InnoScan 710-IR scanner for extended dynamic range signal acquisition at 785 nm.

Scanner fluorescence data were imported into a SuperCurve based Bioinformatics () program.^42–44^ After correcting for local background noise, points indiscernible from background were excluded (signal-to-noise ratio, <2; net fluorescence, <5), and secondary-only signals were subtracted from corresponding slides. Net intensity versus dilution was plotted on a log_2_-log_2_ scale; each local block of samples was fit individually, using interquartile range to exclude outliers outside upper and lower bounds. The slope of the linear portion of the logistic curve was calculated and the line extrapolated back to zero (i.e., the y-intercept), assessing the amount of protein expressed. Total amount of antigen was determined by the y-axis intercept or Y-cept (i.e., by extrapolating the regression line to zero). Here, we express the Y-cept values as log_2_-transformed Y-cept values, which therefore express the total net intensity of the undiluted plasma sample.

### Autoantibody screening

For autoantibody screening, baseline and 3-month post-HWT serum samples from a subset of participants (*n* = 10; all subjects were instructors except for 108 and 109) were analyzed using forward phase human protein microarrays containing immobilized a-SYN, CLDN5, ICAM-1, MBP, and CHRNA7 as service for fee (Abbott, Chicago IL). Microarray fluorescence intensity data were compiled, and the results were analyzed using PAM (Prediction Analysis of Microarrays; Stanford University) to determine only those autoantibody responses that truly sort subjects into their respective disease class. PAM is a technique for class prediction from gene or protein expression data using nearest shrunken centroids.^45^ The method of nearest shrunken centroids identifies subsets of genes or proteins that best characterize each class. The technique is general and can be used in many other classification problems.

### Statistical analysis

Two-way analyses of variance (ANOVAs) were performed for each marker to look for main effects in *Time*, *Group*, and any interactions, in addition to one-way ANOVAs at each time point to look for main effects in *Group* (i.e., instructors vs. students). All data were analyzed using IBM SPSS Statistics software (version 23; IBM Corp., Armonk, NY). Tests were two-tailed with α = 0.05. Scatter plots of log base 2 signal intensity were created for instructors and students at each time point to compare group/time-point median and distribution of relative signal intensity.

## Results

**FIG. 2.**
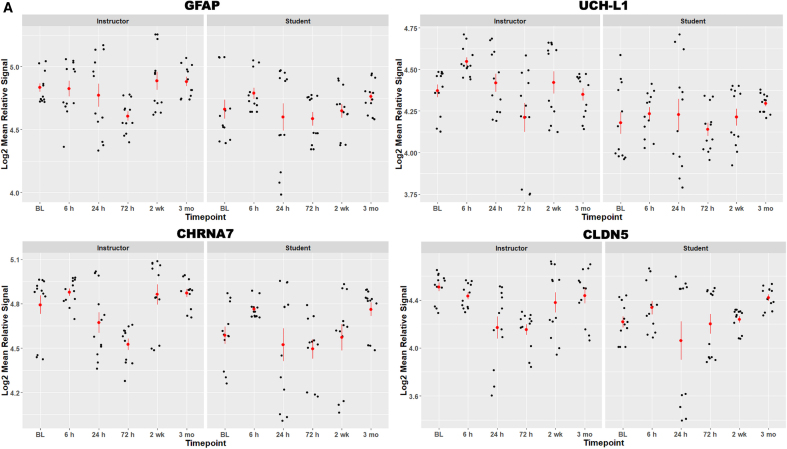

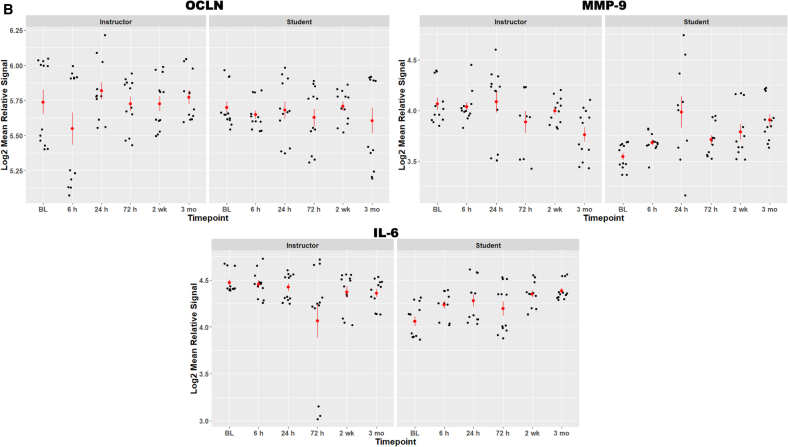
(**A,B**) Scatter plot visualization of protein biomarker data for all persons across time points. Red bars are standard error of the mean. Serum biomarker values are expressed as log_2_ mean relative signal per biomarker. BL, baseline; GFAP, glial fibrillary acidic protein; UCH-L1, ubiquitin carboxy-terminal hydrolase L1; CHRNA7, cholinergic receptor nicotinic alpha 7 subunit; CLDN5, claudin-5; OCLN, occludin; MMP9, matrix metallopeptidase 9; IL-6, interleukin 6.

We found that RSCBEs can alter serum levels of protein biomarkers indicative of astroglial (GFAP), neuronal (UCH-L1 and CHRNA7), and vascular damage (CLDN5 and OCLN) and inflammation (MMP9 and IL-6; Fig. 2; please note that biomarker values are on a log_2_ scale). Serum levels of measured biomarkers were generally higher in the instructor cohort, especially at baseline. This is likely attributable to differences in exposure levels between the RSCBE naïve cohort (i.e., students) and instructors who have experienced multiple RSCBEs during their ongoing assignments. Serum levels of measured markers did not show significant changes over time in the instructor cohort. Conversely, temporal profiles of glial and neuronal markers (i.e., GFAP, UCH-L1, and CHRNA7) appeared to be biphasic in the student cohort. Mean serum levels were elevated at 6 h compared to baseline (phase 1), then slightly reduced at 24 and 72 h before increasing again at the 2-week and 3-month time points (phase 2). Interestingly, vascular and inflammatory markers reached their first peak later in the student cohort at 24 h post-HWT.

The largest differences between students and instructors were found in serum levels of MMP9 and IL-6, markers of extracellular matrix damage and inflammation. Serum levels were substantially higher at baseline in the instructor cohort, indicating an ongoing inflammatory process. In the student group, serum levels of MMP9 and IL-6 increased after RSCBE exposure with a first “spike” at 24 h, a drop at 72 h, and then another increase at 2 weeks and 3 months.

Two-way ANOVAs showed a main effect for *Group* in all tested markers, except OCLN, such that overall serum biomarker levels were higher in instructors than in students; OCLN levels were similar in the two groups. We also found a main effect for *Time* in GFAP, UCH-L1, CHRNA7, and CLDN5, such that the lowest serum biomarker levels were measured at 24 h (followed by 72 h). OCLN levels were lowest at 6 h and 3 months, whereas MMP9 and IL-6 were lowest at baseline and 72 h. The highest biomarker levels in both cohorts were measured at 6 h and 3 months, except for OCLN (highest at 24 h and 2 weeks). MMP9 and IL-6 levels were highest at 24 h and 3 months, respectively. MMP9 and IL-6 were the only two markers with significant Group*Time interactions. Finally, statistically significant differences in serum biomarker levels at each time point (i.e., one-way ANOVAs) are as indicated in Figure 2.

**FIG. 3.**
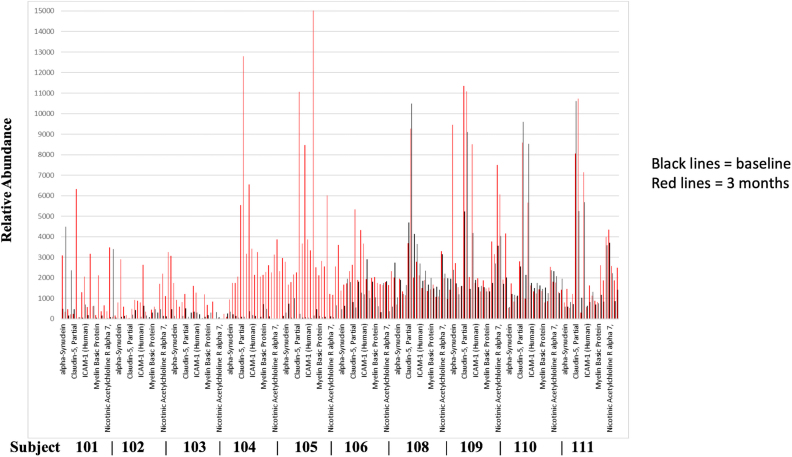
Summary of autoantibody screening against a-SYN, CLDN5, ICAM-1, MBP, and CHRNA7 (screenshot). Data represent baseline autoantibody values (black lines) compared to titers found at 3 months (red lines) from 10 subjects (Instructors = 101–106, 110, and 111; Students = 108 and 109).

We compared sera obtained before and 3 months after RSCBEs for the presence of autoantibodies against a-SYN, CLDN5, ICAM-1, MBP, and CHRNA7 (Fig. 3). Our screening yielded several interesting and potentially important findings. 1) Based on baseline autoantibody levels (black lines), the 10 subjects can be divided into two groups: one with low baseline autoantibody levels (subjects 101–105) and the other with elevated baseline levels (subjects 106–111). 2) Within the first group, there were 2 subjects (104 and 105) whose autoantibody levels were substantially elevated 3 months after exposures, whereas 3 subjects (101–103) had only moderate increases. 3) Autoantibody levels only moderately increased in subjects (106–111) with high initial baseline autoantibody levels. 4) Autoantibody levels against the five selected proteins varied widely, both at baseline and at the 3-month time point, suggesting different antigenicity and/or different circulating serum levels of these five proteins.

## Discussion

Data from this pilot study suggests that RSCBEs in the current configuration of HWT can cause molecular-level changes that can be detected by blood-based protein biomarker analysis. The measured biomarkers represent the main pathobiological changes observed at various post-injury time points by numerous TBI studies.^46–48^ The sampling schedule was selected to cover acute, subacute, and chronic post-exposure time points. However, the time points were also dictated by the HWT schedule and logistics.

Serum levels of measured biomarkers were generally higher in the instructor cohort than in the student cohort, including, importantly, baseline values. Age can play a role given that instructors are older than students, but the likely cause can be the result of the much higher number of subconcussive blasts instructors are exposed to during their 2-year assignments attending numerous HWTs.

We observed an apparent biphasic pattern in the temporal profiles of most markers, especially in students. Similar temporal patterns have been observed in clinical TBI studies that used serial blood sampling (e.g., see a previous work^49^) and are well demonstrated in experimental studies.^34^ This pattern is attributable to two injury processes that ensue post-TBI: primary and secondary injury processes. The primary injury process, caused by the physical impact itself, results in structural damage that is reflected by an initial increase in the serum levels of cellular damage biomarkers (GFAP and UCH-L1).^48,50^ Injury induces a biological response to the damage, the secondary injury process, that is aimed at minimizing damage, restoring functionality, and homeostasis.^48^ The main component of the secondary injury process, albeit still not fully understood, is neuroinflammation. Neuroinflammation initially aids the recovery process by “cleaning up” cellular debris, but if it remains active, it will continue attacking cells, resulting in additional cell damage as indicated by elevated serum levels of inflammatory biomarkers during the subacute and even chronic phase of injury.^48^

Injury to neurons was also indicated by a lasting increase in serum levels of CHRNA7, a nicotinic acetylcholine receptor protein, and by the presence of anti-CHRNA7 autoantibodies 3 months after RSCBE. In the brain, CHRNA7 receptors are localized on GABAergic and glutamatergic terminals in the hippocampus.^12^ Abnormal cholinergic signaling attributable to altered CHRNA7 functionality (e.g., genetic mutations) has been associated with several neuropsychiatric disorders, including schizophrenia, bipolar disorder, impaired cognition and memory, and Alzheimer's disease.^51^ Importantly, CHRNA7 is also expressed by astrocytes, microglia cells, and macrophages and is involved in mediating the cholinergic regulation of various inflammatory conditions, given that activation of CHRNA7 inhibits the production and release of proinflammatory cytokines.^52^

A known inducer of neuroinflammatory response after TBI is vascular injury.^53^ We observed elevated serum levels of CLDN5, an intramembrane protein of endothelial cells,^54^ and a key protein of endothelial tight junction that is involved in regulating BBB permeability. We also found elevated anti-CLDN5 autoantibodies circulating in several participants, which have the potential to cause and/or sustain leakage of the BBB.^54^ Vascular injury triggering a neuroinflammatory process has been hypothesized as the main driver of continuing tissue damage.^55^ Elevation of markers such as CLDN5 and OCLN is likely the interface between primary injury-induced vascular damage and the initiation of secondary injury mechanisms involving neuroinflammation, indicated by elevated IL-6 levels in sera of the student group that persist up to 3 months after RSCBEs. IL-6 is a key mediator of inflammation; it is produced in response to cellular injuries and infections.^56^ IL-6 plays a major regulatory role in coordinating immune response and the host defense mechanism.^57^ In addition to its role as an immune mediator, IL-6 may also modulate neurotransmission, and there is evidence that it plays a role in the pathomechanism of depression.^58^

Another potential interface between RSCBE-induced primary injury and inflammation can be the activation of MMP9. MMP9 is an extracellular protease, which can be released from neurons, glia, and leukocytes after various types of insults.^14^ MMP9 is also a major contributor to inflammatory responses after brain insults; it increases BBB permeability and enables leukocyte migration into the brain.^14,59^ MMP9 has also been involved in the pathogenesis of several autoimmune disorders.^60^ Importantly, chronically elevated levels of MMP9 (e.g., after trauma or stroke) have been implicated in neurodegeneration.^14^

We detected varying levels of circulating autoantibodies against five selected proteins. In some subjects, autoantibody titers appeared higher 3 months after RSCBEs compared to their baseline levels, suggesting that RSCBEs may lead to the activation of B cells and autoantibody production (for review, see a previous work^18^). Several clinical and experimental studies have also demonstrated the presence of autoantibodies against several brain-specific proteins after TBI.^61^ Anti-a-SYN autoantibodies were found in patients with neurodegenerative conditions, primarily Parkinson's disease,^62,63^ and circulating anti-CLDN5 autoantibodies are indicative of altered BBB function.^18,64^ ICAM-1, MBP, and CHRNA7 autoantibodies have been detected in various inflammatory conditions and demyelinating disorders.^65^ Anti-CHRNA7 autoimmunity has been suspected in the pathomechanism of Alzheimer's disease,^66^ and because CHRNA7 is expressed in immune cells, the presence of circulating anti-CHRNA7 autoantibodies has the potential to affect the cholinergic regulation of neuroinflammatory responses.^67,68^

There are several limitations of this study that include a small number of subjects and the absence of controls. In this pilot, the baseline values of each subject served as their own control. However, additional factors, such as age, the exclusively male population, physical stress, previous injuries, and other underlying conditions before and during HWT, could have affected our biomarker data. The higher baseline levels of markers detected in instructors can be affected by age, given that instructors are typically older than students. The results from the autoantibody screening need to be extended to the entire student and instructor cohort, and after the availability of the physical data (“blast-load”), a “dose response” and potential individual “thresholds” can be determined.

This was a highly compartmentalized study; physical parameters (i.e., blast-load) and other biological outcome measures, such as imaging and functional data, will be available pending institutional reviews. The complete I-TAB data set will enable us to assess both within and between individual variability in structural, functional, cognitive, and biomarker levels across time and will help to revise safety protocols if necessary.

In summary, in this pilot study, we found that it is feasible to use blood-based protein biomarker analysis to monitor the effects of RSCBEs in order to ensure safety. Our data indicate that RSCBEs in the current HWT training scenario can result in lasting molecular changes as shown by elevated serum levels of protein biomarkers indicative of vascular injury and inflammation as well as by the presence of autoantibodies against some of the same proteins at the 3-month post-exposure time point. Our findings also highlight the importance of multiple sampling time points and using protein biomarkers of various functional classes for a comprehensive understanding of ongoing disease processes.

## Supplementary Material

Supplemental data

## Authors' Contrbutions

D.V.A. led the protein biomarker study and wrote the study report and manuscript. J.M., R.A., I.-H.L., A.K., and M.E. performed the proteomic analyses, analyzed the data, and created the graphs. L.S.R. led the field study and supervised sample collection. E.B.S. oversaw data analysis. F.L. coordinated the operations of the I-TAB study. J.L.D. designed and led the I-TAB study. E.B.S., F.L., and J.L.D. reviewed and revised the manuscript. A.K. performed the final editing.

## Abbreviations Used

ANOVAs: analyses of variance
a-SYN: alpha-synuclein
BBB: blood–brain barrier
CHRNA7: nicotinic alpha 7 subunit
CLDN5: claudin-5
CTE: chronic traumatic encephalopathy
GFAP: glial fibrillary acidic protein
HWT: heavy weapons training
ICAM-1: intracellular adhesion molecule 1
IL-6: intereukin-6
MBP: myelin basic protein
MMP9: matrix metalloprotease 9
OCLN: occludin
PAM: Prediction Analysis of Microarrays
RPPM: reverse phase protein microarray
RSCBE: repetitive subconcussive blast exposure events
TBI: traumatic brain injury
UCH-L1: ubiquitin C-terminal hydrolase L1
