# Supplementary material for: Blood-Based Biomarkers of Repetitive, Subconcussive Blast Overpressure Exposure in the Training Environment: A Pilot Study
Source: Neurotrauma Rep. 2022 Oct 31;3(1):479–90. doi: 10.1089/neur.2022.0029 (PMC9634979; doi:10.1089/neur.2022.0029)
Supplement: Supplemental data [file Suppl_TableS1.docx]

**Appendix**

**List of protein biomarkers assayed and antibody details for RPPM analysis**. GFAP = glial fibrillary acidic protein; UCH-L1 = ubiquitin carboxyl-terminal hydrolase L1; CHRNA7 = Cholinergic receptor nicotinic alpha 7 subunit; CLDN5 = claudin 5; OCLN = occludin; MMP9 = matrix metalloproteinase 9; IL-6 = interleukin 6.

| **Protein biomarker** | **Primary Ab**  **(vendor, product #, dilution)** | **Secondary Ab**  **(vendor, product #, dilution)** |
| --- | --- | --- |
| GFAP | Abcam, ab7260, 1:1000 | Thermo Fisher, A27041  1:10,000 (Gt anti-Rb) |
| UCH-L1 | Cell Signaling, 11896, 1:100 | Thermo Fisher, A27041  1:10,000 (Gt anti-Rb) |
| CHRNA7 | Abcam, ab10096, 1:40 | Thermo Fisher, A27041  1:10,000 (Gt anti-Rb) |
| CLDN5 | EMD Millipore, ABT45, 1:200 | Thermo Fisher, A27041  1:10,000 (Gt anti-Rb) |
| OCLN | ThermoFisher, 71-1500, 1:100 | Thermo Fisher, A27041  1:10,000 (Gt anti-Rb) |
| MMP9 | Abcam, ab38898, 1:500 | Thermo Fisher, A27041  1:10,000 (Gt anti-Rb) |
| IL-6 | Abcam ab6672, 1:125 | Thermo Fisher, A27041, 1:10,000 (Gt anti-Rb) |
